# Prognostic Effects of Predominant Histologic Subtypes in Resected Pulmonary Adenocarcinomas

**DOI:** 10.4274/balkanmedj.galenos.2019.2019.1.130

**Published:** 2019-10-28

**Authors:** Demet Yaldız, Şeyda Örs Kaya, Kenan Can Ceylan, Arkın Acar, Zekiye Aydoğdu, Soner Gürsoy, Sadık Yaldız

**Affiliations:** 1 Department of Thoracic Surgery, Celal Bayar University School of Medicine, Manisa, Turkey; 2 Clinic of Thoracic Surgery, University of Health Sciences, İzmir Dr. Suat Seren Chest Diseases and Thoracic Surgery Training and Research Hospital, İzmir, Turkey; 3 Clinic of Pathology, University of Health Sciences, İzmir Dr. Suat Seren Chest Diseases and Thoracic Surgery Training and Research Hospital, İzmir, Turkey

**Keywords:** Adenocarcinoma, lung, papillary, prognosis, solid

## Abstract

**Background:**

Predominant histologic subtypes have been reported as predictors of survival of patients with pulmonary adenocarcinoma.

**Aims:**

To evaluate the predictive value of histologic classification in resected lung adenocarcinoma using the classification systems proposed by the International Association for the Study of Lung Cancer, American Thoracic Society, European Respiratory Society, and World Health Organization (2015).

**Study Design:**

Cross-sectional study.

**Methods:**

The histologic classification of a large cohort of 491 patients with resected lung adenocarcinoma (stages I-III) was retrospectively analyzed. The tumors were classified according to their predominant component (lepidic, acinar, papillary, solid, micropapillary, and mucinous), and their predictive values were assessed for clinicopathologic characteristics and overall survival.

**Results:**

The patient cohort comprised 158 (32.2%) patients with solid predominant, 150 (30.5%) with acinar predominant, 80 (16.3%) with papillary predominant, 75 (15.3%) with lepidic predominant, 22 (4.5%) with mucinous, and 5 (1.0%) with micropapillary subtype, and 1 (0.2%) with adenocarcinoma *in situ*. Overall 5-year survival of 491 patients was found to be 51.8%. Patients with lepidic, acinar, and mucinous adenocarcinoma had 70.9%, 59.0%, and 66.6% 5-year survival, respectively, and there was no statistically significant difference between them. Whereas patients with solid, papillary, and micropapillary predominant adenocarcinoma had 41.0%, 40.5%, and 0.0% 5-year survival, respectively. Compared to other histologic subtypes, patients with solid and papillary predominant adenocarcinoma had significantly lower survival than those with lepidic (p<0.001, p=0.002), acinar (p<0.001, p=0.008), and mucinous (p=0.048, p=0.048) subtypes, respectively. The survival difference between patients with solid subtype and those with papillary subtype was not statistically significant (p=0.67).

**Conclusion:**

Solid and papillary histologic subtypes are poor prognostic factors in resected invasive lung adenocarcinoma.

Pulmonary adenocarcinoma is a heterogeneous group of tumors characterized by various predominant histologic subtypes. Identification of prognostic and predictive factors in patients with resected lung adenocarcinoma is essential for risk stratification for further management. In 2011, the International Association for the Study of Lung Cancer, the American Thoracic Society, and the European Respiratory Society proposed a new classification system for lung adenocarcinomas and defined major histological subtypes (acinar, lepidic, papillary, solid, and micropapillary) (1). Additionally, in 2015, the World Health Organization issued a new classification system for lung adenocarcinomas according to their predominant subtypes (2). Since the release of the new classification system, many studies have widely demonstrated the solid and micropapillary predominant subtypes to be associated with poorer overall survival, whereas, the lepidic predominant subtype to have the most favorable outcome (3,4). However, the prognostic effect of the other predominant subtypes has not been clearly described and needs further clarification.

In this study, we analyzed 491 consecutive patients with stage I-III invasive lung adenocarcinoma who underwent surgical resection between 2005 and 2016. Our aim was to elucidate the predictive and prognostic values of predominant subtypes of lung adenocarcinoma.

## MATERIALS AND METHODS

Clinical patient records were retrospectively evaluated to retrieve data of patients who had curatively undergone resection of primary lung adenocarcinoma from 2005 to 2016 (n=534). Patients who underwent neoadjuvant therapy, incomplete resection of metastatic disease, or had any existing nodule at the time of surgery were excluded from the study. The participation rate in the study was 91.9% (491/534).

Before 2010, all patients were screened using contrast-enhanced chest computed tomography, and after 2010, they were additionally screened using positron emission tomography computed tomography to detect unknown metastases. Patients without enlarged lymph nodes and a positron emission tomography-negative mediastinum proceeded directly to surgery. However, patients with enlarged lymph nodes on computed tomography, irresepective of positron emission tomography findings, underwent endobronchial ultrasound transbronchial needle aspiration and/or mediastinoscopy. Patients with N2 disease had received adjuvant chemotherapy and/or radiotherapy.

From 2005 to 2010, serratus anterior muscle-sparing thoracotomy was performed in all patients. Since 2010, nearly 25% of the patients underwent video-assisted thoracoscopic surgery.

**Figure 1:**
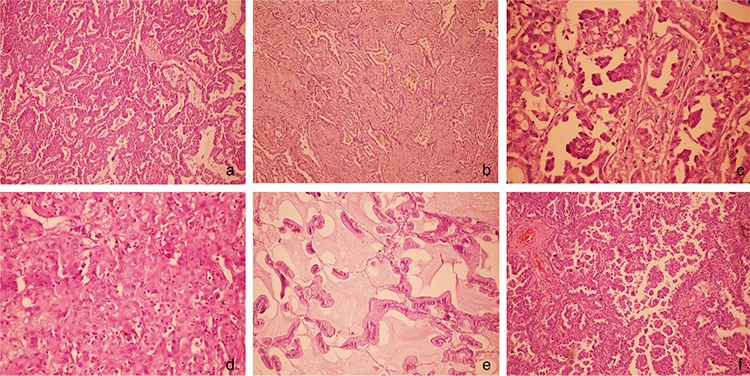
**a-f.** Microphotographs showing, acinar (H&E x200) (a), lepidic (H&E x100) (b), papillary (H&E x200) (c), solid (H&E x200) (d), mucinous (H&E x200) (e), micropapillary (H&E x200) (f).

Medical records of 491 patients were screened for age, sex, smoking history, comorbidity, type of resection, histologic subtype, and pathologic tumor-node-metastasis stage according to the 7^th^ edition of the lung cancer staging system. For the first 2 years, patients were followed up at 3-month intervals and thereafter at 6-month intervals. Mean duration of clinical follow-up was 44.25 months (minimum/maximum: 2-152 months). The date of death was found from the medical records and verified by a software program linked to the national population registration system. Pathological slides were reviewed by pathologists and classified according to the predominant histologic subtype as defined by the International Association for the Study of Lung Cancer/American Thoracic Society/European Respiratory Society (1) and the recent World Health Organization classification systems. On pathological examination, adenocarcinoma with a single-row organization using the alveolar roof was defined as a lepidic pattern, the one that formed circular glandular structures including lumen as an acinar pattern, structures containing fibrovascular core into the lumen as papillary pattern, that containing glandular-cell groups that develop into lumen without fibrovascular core as micropapillary pattern, and that containing layered-cell groups without glandular and papillary structures as solid pattern. In addition, any growth pattern containing abundant intracytoplasmic mucin was defined as mucinous adenocarcinoma. Because most adenocarcinomas are heterogeneous, the dominant model is based on the proportions in the samples (Figure 1). Finally, we compared 5-year survival of predominant histologic subtypes in similar stages and in similar tumor and node status.

This study was approved by the ethical committee of the university (Date: 29.01.2018, No: 49109414-806.02.02). Written informed consent was obtained from all the patients.

### Statistical analysis

Statistical evaluation was conducted using IBM SPSS version 21.0 software (IBM Corp., Armonk, NY, USA). Patient survival was expressed by actuarial analysis according to the method of Kaplan–Meier, and differences in survival were assessed by the log rank test in univariate analysis. A multivariate analysis of variables was performed using the Cox proportional odds regression model. For all statistical analyses, type I error was considered as <0.005. Sample-size calculations were performed using PASS 2008 program. For the variables including age, sex, histologic type, solid and papillary component, pathological stage, node and tumor status, pleural invasion, and extent of resection, log hazard ratio in the Cox regression analysis was used. The result of power analysis for 491 patients was calculated as 100% at α=0.05 significant level.

## RESULTS

### Clinicopathologic characteristics of patients

In total, 491 patients who underwent resection of lung adenocarcinoma were included in this study. There were 410 (83.5%) men and 81 (16.5%) women. The mean age was 60.2 years (range, 26-82 years). Lobectomy was performed in 77.8% (n=382), sublobar resections in 3.7% (n=18), bilobectomy in 8.6% (n=42), and pneumonectomy in 10% (n=49) of the patients. The 30-day mortality for lobectomy and pneumonectomy was 1.0% (5 patients) and 1.8% (9 patients), respectively. At least one morbidity occurred in 137 (28%) of 491 patients.

### Survival analyses

**Figure 2:**
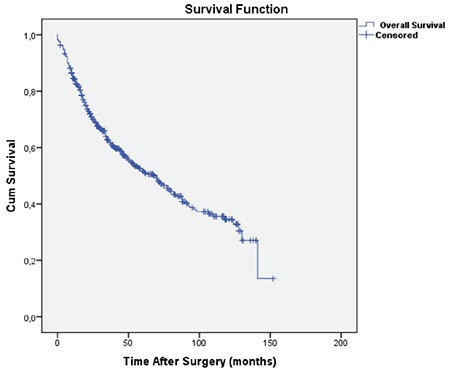
The overall survival curve for all patients (Mean survival; 75.1±3.2 months).

**Figure 3:**
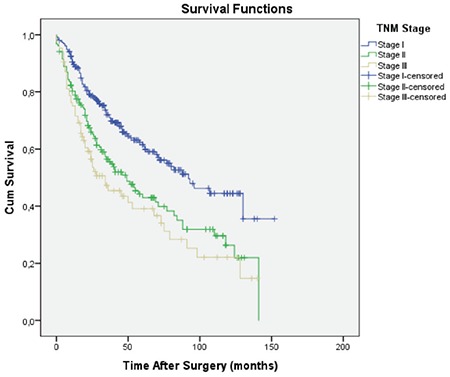
The overall survival curves according to sages. Mean survival in patients with sage I, II, and III were 89.6, 64.7, 55.2 months, respectively.

Overall 5-year survival of 491 patients was found to be 51.8% (Figure 2). The number of cases in stages I, II, and III were 253 (51.5%), 153 (31.2%), and 85 (17.3%), respectively, and 5-year survival rates were 61.5%, 42.9%, and 39.1%, respectively (Figure 3).

Of all patients, 158 (32.2%) exhibited solid predominant, 150 (30.5%) acinar predominant, 75 (15.3%) lepidic predominant, 80 (16.3%) papillary predominant, 22 (4.5%) mucinous, and five (1.0%) micropapillary subtype, and one (0.2%) adenocarcinoma* in situ*. The clinicopathologic variables and their effects on survival in all 491 patients are shown in Table 1.

### Univariate and multivariate analyses

Univariate analysis showed that age ≥60 years (p=0.006), male sex (p=0.007), papillary vs lepidic (p=0.002), papillary vs acinar (p=0.008), papillary vs mucinous (p=0.048), solid vs lepidic (p<0.001), solid vs acinar (p<0.001), solid vs mucinous (p=0.048), presence of solid and/or papillary component (p<0.001), pathologic stage >I (p<0.001), tumor status >T1 (p=0.001), node involvement (p<0.001), pleural invasion (p=0.002), and pneumonectomy (p<0.001) were significantly associated with poor survival. On the other hand, multivariate analysis showed that age (OR=1.49, 95% CI: 1.14-1.94, p=0.004), solid and/or papillary component (OR=1.84, 95% CI: 1.35-2.51, p<0.001), node involvement (OR=1.76, 95% CI: 1.21-2.56, p=0.003), pleural invasion (OR=1.46, 95% CI: 1.04-2.06, p=0.029), and pneumonectomy (OR=1.65, 95% CI: 1.13-2.40, p=0.009) were independent predictors of 5-year survival (Table 1).

**Figure 4:**
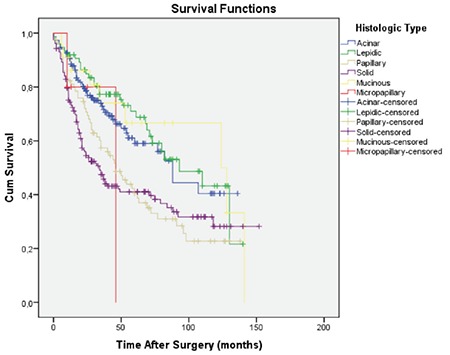
The overall survival curves according to the histopathological subtypes. Mean survival for mucinous, lepidic, acinar, solid, papillary, and micropapillary were 97.8, 89.0, 82.7, 65.0, 61.2, and 38.8 months, respectively.

Patients with lepidic, acinar, and mucinous adenocarcinoma had 70.9%, 59.0%, and 66.6% 5-year survival, respectively, with no statistically significant difference between them. Whereas, patients with solid, papillary, and micropapillary predominant adenocarcinoma had 41.0%, 40.5%, and 0.0% 5-year survival, respectively (Figure 4). Compared to other histologic subtypes; patients with solid and papillary predominant adenocarcinoma had significantly lower survival than those with lepidic (p<0.001, p=0.002), acinar (p<0.001, p=0.008), and mucinous (p=0.048, p=0.048) subtypes, respectively. The survival difference between the patients with solid subtype and those with papillary subtype was not statistically significant (p=0.668). Micropapillary predominant pattern had worse prognosis and 5-year survival was zero. Lepidic predominant adenocarcinoma showed a considerably better survival than all other growth patterns (Table 2).

Overall solid and/or papillary component was present in 327 patients (66.6%) with a 5-year survival of 43.4%, and the solid and/or papillary component-free 164 patients (33.4%) had 67.7% 5-year survival (p<0.001).

**Figure 5:**
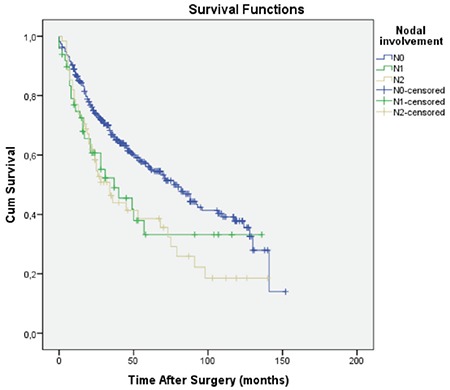
The survival curves according to the nodal stages. Mean survival for N0, N1, and N2 disease were 79.9, 60.6, and 54.4±7.0 months, respectively.

Moreover, N0 status was detected in 381 (77.6%), N1 lymphatic invasion in 49 (10%), and N2 lymphatic invasion in 61 (12.4%) patients. The 5-year survival of N0, N1, and N2 patients was found to be 56.1%, 33.2%, and 38.6%, respectively (Figure 5). Correlation of subtype patterns with the tumor-node-metastasis status and 5-year survival are summarized in Table 2.

Visceral pleural invasion was observed in 103 (19%) patients, and 5-year survival was found to be 29.5%.

## DISCUSSION

Since the International Association for the Study of Lung Cancer/American Thoracic Society/European Respiratory Society classification has been proposed, many studies have investigated the correlations among the histologic subtypes and patient prognosis in pulmonary adenocarcinoma (4,5,6). In this study, we reviewed 491 patients with completely resected invasive lung adenocarcinoma for their clinicopathologic characteristics, prognostic factors, overall survival, and survival-associated histologic subtypes as well as their predictive value for prognosis.

A variety of frequencies of the predominant subtypes of invasive adenocarcinomas have been reported in the literature. In a cohort study, in stage I lung adenocarcinoma, the frequencies of lepidic, acinar, papillary, micropapillary, and solid predominant subtype were reported as 5.6%, 45.1%, 27.8%, 2.3%, and 13.0%, respectively (4). Warth et al. (5) had stated that the frequencies of lepidic, acinar, papillary, micropapillary, and solid predominant subtypes were 8.1%, 42.5%, 4.7%, 6.8%, and 37.6%, respectively, in stage I-IV adenocarcinomas. In our study, the frequencies of predominant patterns were 15.3% for lepidic, 30.5% for acinar, 16.3% for papillary, 1.0% for micropapillary, 32.2% for solid, and 4.5% for mucinous adenocarcinoma.

There is an increasing number of data confirming that solid subtype correlates with poor prognosis (7,8,9,10). In our study, patients with solid as well as papillary predominant adenocarcinoma had significantly worse survival than those with lepidic (p<0.001, p=0.002), acinar (p<0.001, p=0.008), and mucinous (p=0.048, p=0.048) subtypes, respectively, and the survival difference between the patients with solid subtype and those with papillary subtype was not statistically significant (p=0.67). Although some studies have reported somewhat better survival rates for patients with papillary predominant tumors (11), our results are consistent with those of Warth et al. (5) who found similar survival rates for patients with papillary and solid subtypes. Warth et al. (5) also suggested that the worse survival of patients with papillary subtype would probably be a result of different ethnic and geographical patterns. In a study on stage I cases only, Yoshizawa et al. (4) reported similar survival rates for patients with lepidic, acinar, and papillary predominant adenocarcinoma; whereas, in our study, papillary subtype also had significantly worse prognosis when compared to lepidic and acinar subtypes in stage I patients, respectively (p=0.001, p=0.04). Generally, patients with pulmonary mucinous adenocarcinoma are known to have poor overall survival when compared with patients with other subtypes (4,12,13). In our study, we had 22 (4.5%) patients with mucinous adenocarcinoma with a 5-year survival rate of 66.6%. This survival rate is contradictory with the findings of the abovementioned studies. However, recently, Shim et al. (14) showed that the survival of patients with invasive mucinous adenocarcinomas can be compared with those with other predominant subtypes. Finally, Warth et al. (5) reported that patients with invasive mucinous adenocarcinoma did better than most patients with adenocarcinoma. We also assume that these diversities are probably due to the ethnic and geographical differences as suggested by Warth et al. (5).

We had only five patients with micropapillary adenocarcinoma, and they had worse prognosis with a 5-year survival being zero. Consistent with our results, Yoshizawa et al. (15) and Tsubokawa et al. (16) also reported a 0% 5-year survival. Tsubokawa et al. (16) reported micropapillary subtype as an independent predictive prognostic factor even in stage IA patients. They also suggested that these patients may require adjuvant therapy regardless of the stage.

Visceral pleural invasion (p=0.002) was significantly associated with poor survival in our study. It was 10.7% in lepidic subtype; whereas, in papillary, acinar, solid, and micropapillary subtype, it was 21.3%, 24%, 22.7%, and 40%, respectively. Hung et al. (9) stated that visceral pleural invasion was more common in micropapillary and solid predominant groups and less frequent in the lepidic predominant group.

Nodal metastasis rates (N1 + N2) vary by predominant subtype. In our study, lymph node metastases were significantly associated with micropapillary subtype (40%). The other subtypes showed similar rates of nodal metastases (solid 23.4%, acinar 22.7%, papillary 22.5%, and lepidic 18.7%). Travis et al. (1) reported less frequent nodal metastasis in lepidic adenocarcinoma (7%) than papillary (43%), acinar (47%), solid (51%), and micropapillary adenocarcinoma (76%).

Our study has some limitations due to its retrospective nature and single-center design. Secondly, we could not take EGFR or KRAS mutation status into consideration in our analyses.

In conclusion, solid and papillary predominant adenocarcinoma had significantly worse prognosis, and more attention should be paid to these aggressive lung adenocarcinoma subtypes. Even the other subtypes (lepidic, acinar, and mucinous) have an adverse solid and/or papillary component effect on survival. Although stage was a powerful predictor of survival, International Association for the Study of Lung Cancer/American Thoracic Society/European Respiratory Society and World Health Organization classifications are also efficient predictors of patient survival and should be considered in the treatment planning of pulmonary adenocarcinomas. The poor prognostic group of solid, papillary, and micropapillary subtype may be candidates for adjuvant therapy even in the earlier stages of disease, but further studies are warranted.

## Figures and Tables

**Table 1:**
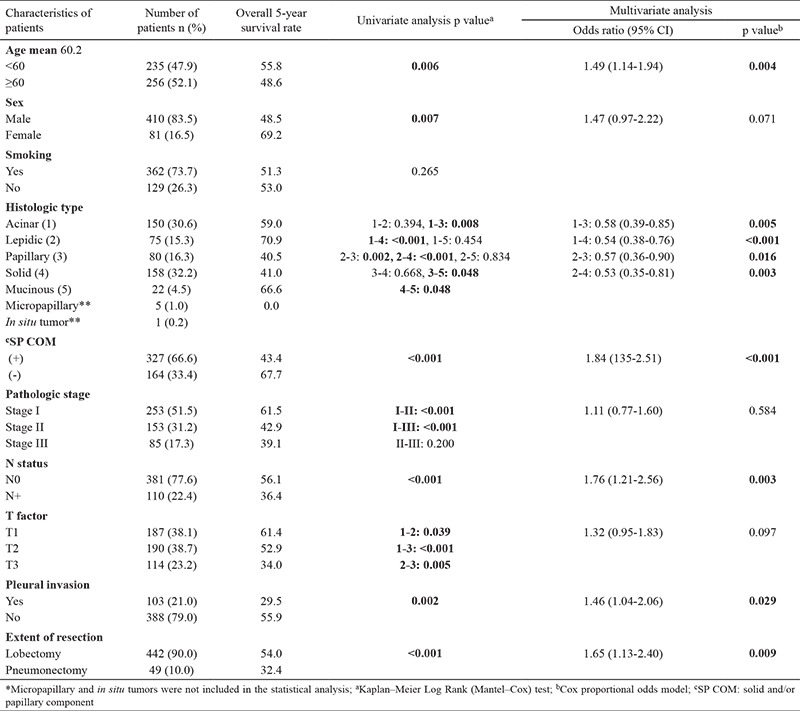
Clinicopathologic variables and their effects on survival in 491 patients

**Table 2:**
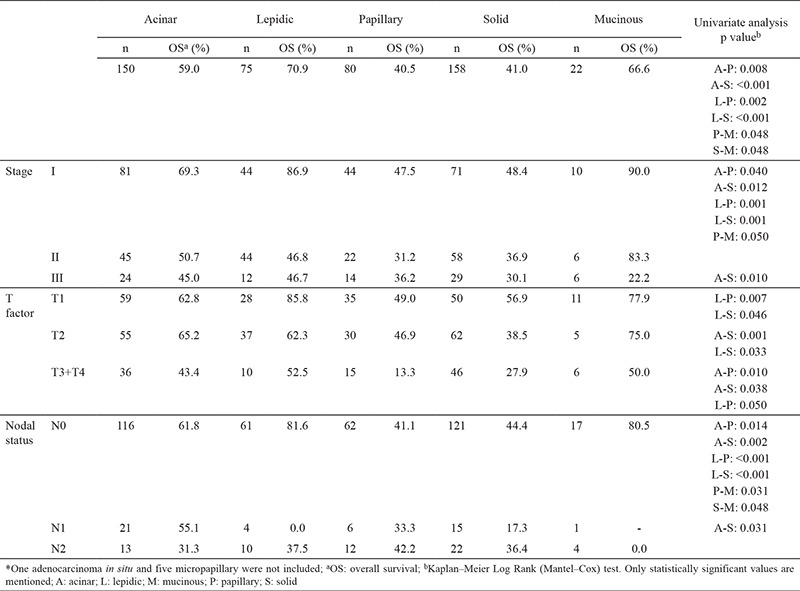
Correlation of subtype patterns with tumor-node-metastasis status and 5-year survival
